# Cisplatin-Induced Apoptosis Inhibits Autophagy, Which Acts as a Pro-Survival Mechanism in Human Melanoma Cells

**DOI:** 10.1371/journal.pone.0057236

**Published:** 2013-02-20

**Authors:** Barbara Del Bello, Marzia Toscano, Daniele Moretti, Emilia Maellaro

**Affiliations:** Department of Pathophysiology, Experimental Medicine and Public Health, Istituto Toscano Tumori, University of Siena, Siena, Italy; Enzo Life Sciences, Inc., United States of America

## Abstract

The interplay between a non-lethal autophagic response and apoptotic cell death is still a matter of debate in cancer cell biology. In the present study performed on human melanoma cells, we investigate the role of basal or stimulated autophagy in cisplatin-induced cytotoxicity, as well as the contribution of cisplatin-induced activation of caspases 3/7 and conventional calpains. The results show that, while down-regulating Beclin-1, Atg14 and LC3-II, cisplatin treatment inhibits the basal autophagic response, impairing a physiological pro-survival response. Consistently, exogenously stimulated autophagy, obtained with trehalose or calpains inhibitors (MDL-28170 and calpeptin), protects from cisplatin-induced apoptosis, and such a protection is reverted by inhibiting autophagy with 3-methyladenine or *ATG5* silencing. In addition, during trehalose-stimulated autophagy, the cisplatin-induced activation of calpains is abrogated, suggesting the existence of a feedback loop between the autophagic process and calpains. On the whole, our results demonstrate that in human melanoma cells autophagy may function as a beneficial stress response, hindered by cisplatin-induced death mechanisms. In a therapeutic perspective, these findings suggest that the efficacy of cisplatin-based polychemotherapies for melanoma could be potentiated by inhibitors of autophagy.

## Introduction

Macroautophagy, commonly referred to as autophagy, is a well-conserved, physiologically controlled self-consuming process through which cytoplasmic components (e.g. damaged organelles, macromolecular aggregates of long-lived proteins, and microbes) are sequestered in double-membrane autophagosomes and subsequently degraded by lysosomal fusion. This catabolic process, by recycling macromolecules, contributes to maintain cellular homeostasis and acts as a housekeeping, survival mechanism in different harmful conditions, including starvation, ER stress and infection. However, an extensive activation of autophagy, hampering cell recovery, can culminate in a peculiar mode of cell demise, classified as autophagic (or type II) cell death [1], [2].

With the identification of autophagy as a cell death program alternative to apoptosis, its contribution to tumorigenesis has been explored as well. Differently from the unambiguous role of apoptosis in tumor suppression, the relation between autophagy and cancer appears to be multifaceted and intricate, essentially for two aspects. First, the autophagic process can lead to opposite end-points (survival or death); second, either down-regulation or mild stimulation of autophagy could benefit tumor cells, depending on the stage of cancer development and on its specific demands. In fact, down-regulation of autophagy can be useful in favourable metabolic conditions, when the predominance of protein synthesis over protein degradation is required for sustaining cell growth; on the other hand, in an established tumor, a mild autophagy activation may provide a mechanism through which cancer cells overcome unfavourable metabolic conditions (including hypoxia and limited nutrients), as occurring in poorly vascularized tumors [3], [4].

The picture is even more complex when tumor cells are stressed by therapeutic drugs which stimulate apoptosis. Possibly depending on the tumor cell type used or the autophagy “source” (basal or exogenously stimulated), controversial views on the role of autophagy in tumor therapy have emerged in the literature: it has been suggested that the autophagic response observed in cells treated with diverse cytotoxic drugs can be a rescue mechanism that protects tumor cells from apoptosis or, alternatively, it can be a mechanism contributing to (apoptotic) cell death [5]–[7]. At the best of our knowledge, no exhaustive data are available about the role of autophagy in cisplatin-treated human melanoma cells. The topic is particularly relevant, since cisplatin is currently used in poly- and bio-chemotherapy regimens, which, however, remain unsatisfactory to treat metastatic melanomas.

Against this background, the present study, performed in human melanoma cells sensitive to cisplatin, was aimed to investigate the interplay between the drug-induced apoptosis and the basal or stimulated autophagic process. The contribution of conventional calpains in such an interplay was also explored. Calpains are a family of Ca^++^-dependent non-lysosomal cysteine proteases, including numerous gene (and splicing variants) products [8]–[11], both ubiquitous and tissue-specific isoforms. Calpain 1 and calpain 2 (conventional calpains) are the best characterized ubiquitous isoforms, proved to be involved in diverse pathophysiological cellular events, such as apoptotic death of tumor cells [8], [10] and autophagy [12]–[15]. Concerning apoptosis, in cisplatin-treated melanoma cells, we have previously demonstrated [16] that the pharmacological inhibition of calpains, which are early activated, protects from apoptotic cell death through a p53-dependent mechanism.

In the present study, we demonstrate that *i)* cisplatin-induced death machinery inhibits the basal autophagic process in melanoma cells, as a further tool contributing to cell demise, and *ii)* autophagy exogenously induced by calpains inhibitors or by the calpain-unrelated compound, trehalose, acts as a pro-survival response against cisplatin cytotoxicity.

## Materials and Methods

### Cell cultures, RNA interference, and treatments

Human metastatic melanoma cells Me665/2/21 (henceforth called Me21) (kindly provided by Dr. Zunino and Dr. Supino, Istituto Nazionale Tumori, Milan) [17] and human metastatic melanoma cells HT-144 (from ATCC) were cultured in RPMI-1640 medium (Sigma, R5886) containing 10% heat-inactivated foetal bovine serum (Invitrogen, 10270), 50 mg/L gentamycin (Sigma, G1264), 2 mM L-glutamine (Sigma, G6392), at 37°C, in a humidified atmosphere with 5% CO_2_. Human melanoma cells A375 (from ATCC) were cultured in Dulbecco's modified Eagle's medium (DMEM) with 4.5 g/L glucose (Sigma, D5671), containing 5% heat-inactivated foetal bovine serum. For routine reseeding before reaching confluence and for experiments, cells were harvested with TrypLE™ Express Stable Trypsin-Like Enzyme with Phenol Red (Invitrogen, 12604-013).

For experiments, the seeded cells were rested overnight and then treated in fresh medium with the following compounds: cisplatin (20 µM for Me21 and A375 cells, and 15 µM for HT-144 cells) (Sigma, P4394) at the constant *ratio* of 0.12 µmoles/10^6^ cells, inhibitors of calpain 1 and 2, MDL-28170 (Z-Val-Phe-aldehyde) (Sigma, M6690) and calpeptin (Z-Leu-norLeu-aldehyde) (Calbiochem, 03-34-0051) (both 40 µM), trehalose (100 or 60 mM) (Sigma, T0167), the caspase-3/-7 inhibitor ac-DEVD-CHO (ac-Asp-Glu-Val-Asp-aldehyde) (50 µM) (Alexis, ALX-260-030-M001), the rapamycin ester temsirolimus (CCI-779, 100 nM) (Wyeth-Ayerst, a gift from M.C.), and the autophagy inhibitor 3-methyladenine (3-MA, 4 mM) (Sigma, M9281), all added at the culture medium at 0 time. The autophagic flux blockers chloroquine (30 µM) (Sigma, C6628) or bafilomycin A1 (200 nM) (Sigma, B 1793) were added 2 hours before cell harvesting. All inhibitors used have been preliminarily tested, in order to choose the lowest effective concentrations giving no or very minor toxicity on control cells.

In a separate set of experiments, in order to down-regulate *ATG5* gene expression, A375 melanoma cells were transfected with ATG5 siRNA. The specific siRNA (sequence CAA CUU GUU UCA CGC UAU A) and the scrambled control siRNA were obtained from Qiagen. Briefly, A375 cells were seeded into six-well plates in complete DMEM medium. The day after, the cells were transfected with 5 nM scrambled siRNA or with 5 nM ATG5 siRNA using HiPerFect transfection reagent (Qiagen, 301704), according to the manufacturer's instructions. After 24 hours of siRNAs transfection, different compounds were added to the cells in fresh medium for further 24 h. The efficacy of *ATG5* down-regulation was assessed by means of Real Time-PCR and Western blot.

At the end of the experiments, we harvested and analyzed the population of still adhering cells together with or separately from the population of detached cells, depending on the investigations to be performed, as specified in sample labels and in figure legends.

### Assessment of apoptosis

Apoptosis was evaluated as percentage of detached cells on total cells and as caspase-3/-7 enzymatic activity. Both adhering and floating cell suspensions were separately counted in a Bürker chamber. The percentage of detached cells has been reliably used as a quantitative indication of apoptotic cell death, since in cisplatin-treated melanoma cells we have previously demonstrated that cell detachment strictly correlates with biochemical and morphological hallmarks of apoptosis [16], [18]. In particular, cell detachment quantitatively mirrors the amount of shrinking nuclei with condensed and fragmented chromatin, the hypodiploid DNA content, the caspase-3/-7 and caspase-9 processing and enzymatic activity, and the caspase-dependent proteolysis of nuclear PARP and lamin B.

### Fluorimetric assay for caspase-3/-7 activity

Caspase-3/-7 enzymatic activity has been measured essentially as previously described [18]. Briefly, the harvested (adhering *plus* floating) cells were washed with ice-cold PBS, and resuspended (10^6^ cells/100 µl) in the following lysis buffer: 20 mM Hepes-NaOH, pH 7.5, containing 10% sucrose, 0.1% CHAPS, 0.2% NP-40, 1 mM EDTA, 5 mM DTT, 1 mM PMSF, and protease inhibitor cocktail (Sigma-Aldrich, P8340). After 30 minutes on ice, cells were sonicated for 10 s (Vibracell Sonicator; amplitude 60, 25 W), centrifuged at 10,000 *g* for 30 s, and the supernatant stored at −80°C until used. Protein concentration was determined using the Bradford method (Sigma, B6916). Cell lysates (50 µg/ml of protein) were incubated with the synthetic substrate ac-Asp-Glu-Val-Asp-7-amido-4-methylcoumarin (ac-DEVD-AMC, 50 µM) (Alexis, ALX-260-031-M001), in the following assay buffer: 100 mM Hepes-NaOH, pH 7.5, containing 10% sucrose, 0.1% CHAPS, 0.1% NP-40, 1 mM EDTA, 5 mM DTT, 1 mM PMSF, and protease inhibitor cocktail. Cleavage of the fluorogenic substrate was monitored by AMC release, at 37°C, in the Multilabel Counter Reader Victor3 (PerkinElmer) (excitation/emission wavelenghts: 380/460 nm), and the cleavage rate was determined in the linear portion (30–50 min) of the progress curve. Caspase-3/-7 activity was expressed as Arbitrary Units of Fluorescence (AUFs)/min/mg protein.

### Fluorimetric assay for calpain activity

The enzymatic activity of conventional calpains was measured in cell lysates, essentially according to Mallya *et al.* [19]. Briefly, cell lysates (150 µg protein/ml) prepared as above described were incubated with the synthetic substrate N-Succinyl-Leu-Tyr-7-amido-4-methylcoumarin (suc-LY-AMC, 200 µM) (Alexis, ALX-260-054-M005) in the following assay buffer: 50 mM TRIS-HCl pH 7.5, 50 mM NaCl, containing 1 mM EDTA, 1 mM EGTA, 0.1% CHAPS, 5 mM β-mercaptoethanol, 1 mM PMSF, 1 µg/ml pepstatin A, 5 µg/ml aprotinin, and 5 mM CaCl_2_ freshly added. The cleavage of the fluorogenic substrate was monitored by AMC release, at 37°C, in Multilabel Counter Reader Victor3 (PerkinElmer) (excitation/emission wavelenghts: 380/460 nm), and the cleavage rate was determined in the linear portion (30–50 min) of the progress curve. Calpain activity was expressed as Arbitrary Units of Fluorescence (AUFs)/min/mg protein.

### Western blot analysis

Aliquots (30–60 µg protein) of cell lysates, as prepared for caspase activity assay, were subjected to SDS-PAGE on 4–15% or 10% Mini-Protean TGX Precast gels (Bio-Rad, 456–1091), for 30 minutes at 200 V, followed by blotting to 0.45 or 0.22 µm nitrocellulose membrane (AppliChem, A5239) for 30 minutes at 200 mA, and blocked in 10% non-fat milk in PBS-Tween 0.05% (PBST). Membranes were probed overnight at 4°C with the following antibodies diluted in PBST-1% non-fat milk: anti-LC3B (microtubule-associated protein 1 light chain 3 B) (Sigma, L7543), anti-Atg14 (Sigma, A6358), anti-Beclin-1 (Santa Cruz Biotechnology, SC-11427). After three washings with PBST, the membrane was incubated with horseradish peroxidase-conjugated secondary antibodies (goat-anti-rabbit, Sigma, R4880, or goat-anti-mouse, Sigma, A5420) diluted in PBST-1% non-fat milk at room temperature for 1 h. Protein bands were visualized by an enhanced chemiluminescence reaction system (Super Signal West Pico, Thermo scientific, 34080) according to manufacturer's instructions, and membranes were then developed on Hyperfilm ECL (Amersham GE Healthcare, 28906835). To assess the autophagic process more accurately, in selected experiments Western blot analysis of LC3 was performed in cells co-treated with autophagic flux blockers chloroquine or bafilomycin A1; blocking the last critical step of autophagy, i.e. clearance of LC3-II through lysosomal degradation, allows to record the actual time-dependent accumulation of autophagic vesicles at the time point of blockage [20]. For these samples, half protein has been loaded in the gel, to avoid the extremely intense LC3-II bands due to accumulation of autophagosomes.

Western blot protein bands were quantified by densitometric analysis (image processing software ImageJ). Before adding primary antibodies, equal gel loading, quality control, and transfer efficiency among samples were assessed by reversible Ponceau S protein staining on membrane. The densitometric values corresponding to a wide range of molecular weight inside the Ponceau stained lanes were used to normalize the expression of protein of interest [21].

### RNA extraction and Real-Time PCR analysis

RNA was extracted with RNeasy Mini Kit (Qiagen, 74104), according to the manufacturer instructions. *ATG5* expression level (mRNA) was measured by Real-Time PCR, using StepOnePlus (Applied Biosystems) and SYBR GreenER qPCR Supermix Universal (Invitrogen, 11762-100), and normalized to the endogenous reference gene *β-actin* (see Table 1 for the primers used).

**Table 1:** Primers used for Real-Time PCR analysis of ATG5 expression.

Primer	Sequence (5′→3′)
5′-ATG5	TCTGCACTGTTCATCTAAGGATGCA
3′-ATG5	TCCGATTGATGGCCCAAAACTGGT
5′-β-Actin	CAGAGCCTCGCCTTTGCCGATCC
3′-β-Actin	GACGACGAGCGCGGCGATATCA

The thermocycling conditions were: 10 minutes at 95°C, followed by 40 cycles of denaturation (95°C for 20 s) and 60°C for 1 minute, for annealing and extension. The expression levels were calculated with 2^−ΔΔc(t)^ method, and compared to that of scrambled siRNA-treated cells and untreated cells (to the latter an arbitrary expression value of 1 was assigned).

### LDH release assay

4×10^5^ cells were seeded in 6-well plates in 2.5 ml of complete culture medium; after an overnight resting, cells were treated with different compounds. The release of intracellular enzyme lactate dehydrogenase (LDH) in the culture medium was evaluated by using LDH-P Kit (Sclavo Diagnostic, 82711), according to the manufacturer's instructions, with modifications. An equal amount (40 µl) of cell-free culture medium was collected for each sample, and LDH activity was measured in a microplate reader (Victor3, Perkin-Elmer). Absorbance was recorded over a 0.5–8-min period, and the relative **Δ**Abs/min was normalized for cell number of each sample. The results are expressed as **Δ**Abs/min/10^6^ cells.

### MDC staining of autophagic vacuoles

Autophagic vacuoles were stained with the autofluorescent agent monodansylcadaverine (MDC) (Sigma, 30432) [22]. Melanoma cells were grown overnight on coverslips, then treated for 24 hours with different autophagy inducers, with or without 3-MA. Cells were then rinsed twice with PBS, and exposed to freshly prepared MDC (50 µM in RPMI-1640 medium) at 37°C, for 10 minutes in the dark; after washing with PBS, cells were fixed with 4% paraformaldehyde. Slides were observed under a fluorescence microscope (Nikon Eclipse Ti, Japan) (excitation/emission wavelengths: 380/420 nm, barrier filter 450 nm). Images were captured with a CCD camera and imported into NIS-Elements Imaging software. The same Imaging software was used to measure the mean fluorescence intensity/cell. At least 200 cells were scored for each sample in each experiment.

### Statistical Analysis

Results are presented as means ± S.E. The statistical significance of the differences among the groups was determined by the Student's *t*-test.

## Results

### Cisplatin inhibits the basal autophagy in Me21 melanoma cells

**Figure 1:**
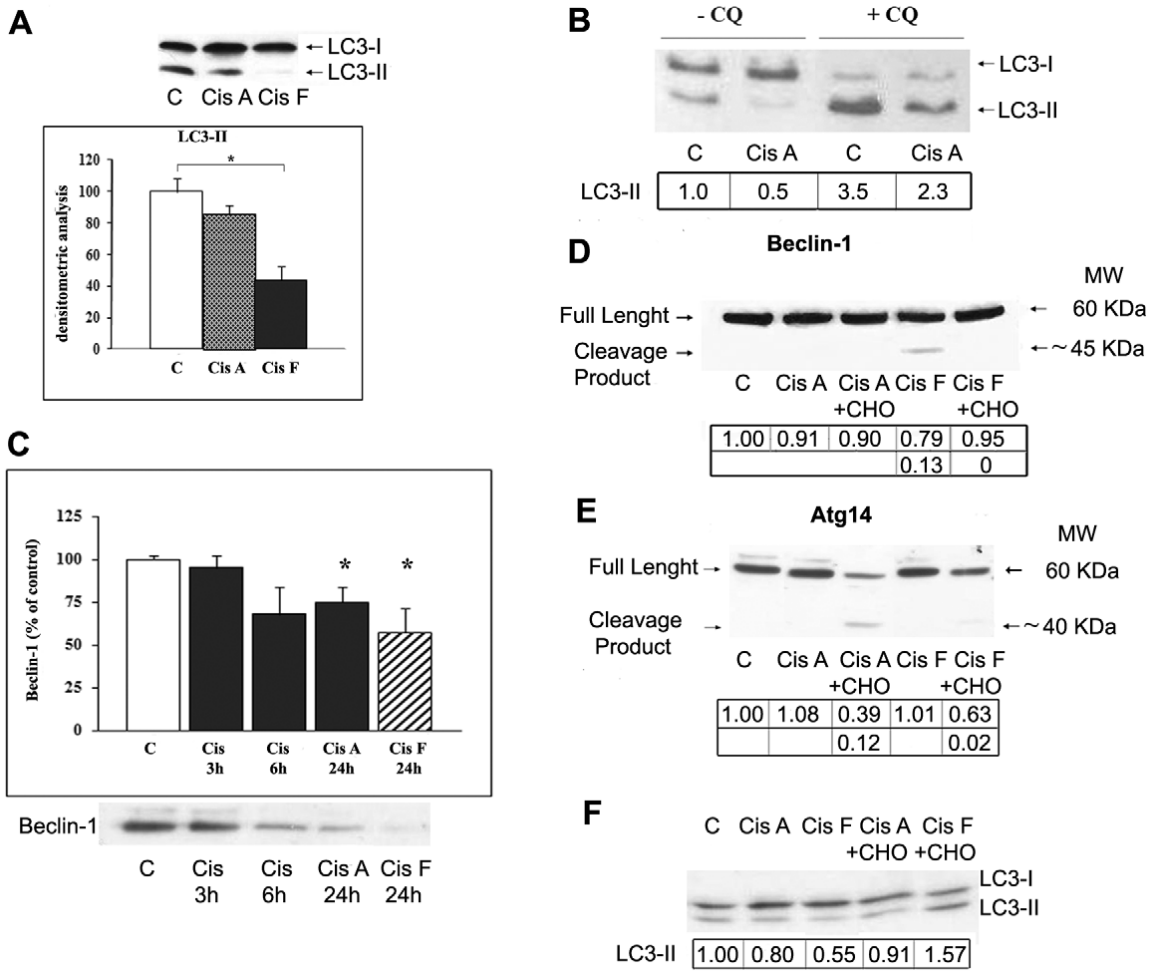
Cisplatin treatment affects autophagy-related proteins and inhibits basal autophagy in Me21 cells. Me21 melanoma cells were untreated (C) or treated with 20 µM cisplatin; after 24 hrs, still viable adhering cells (Cis A) were harvested and analyzed separately from floating dead cells (Cis F). (**A**) Densitometric analysis of LC3-II of 8 experiments is presented as means ± S.E. *P<0.05. Western blot analysis of a typical experiment is shown. (**B**) Western blot analysis of LC3 performed in cells treated with cisplatin for 18 hours *plus* 30 µM chloroquine (CQ) for further 2 hours, in order to block the autophagic flux. In CQ-treated cells half protein has been loaded. A typical experiment out of 3 is shown, and densitometric analysis is reported. (**C**) Down-regulation of Beclin-1 in Me21 melanoma cells at different time-points of cisplatin treatment; densitometric analysis of Western blots is reported as means ± S.E. of 3 to 9 experiments; Beclin-1 level is expressed as percentage of control cells at 0 time. *P<0.05, compared to control cells at 0 time. A typical experiment is shown. (**D**) Beclin-1 proteolysis after 24 hours of cisplatin treatment, in absence or presence of the caspase-3/-7 inhibitor ac-DEVD-CHO (CHO); Western blot and densitometric analysis of a typical experiment out of 3 are reported. (**E**) Atg14 proteolysis after 24 hours of cisplatin treatment, in absence or presence of ac-DEVD-CHO; Western blot and densitometric analysis of a typical experiment out of 3 are reported. (**F**) LC3-II levels after 24 hours of cisplatin treatment, in absence or presence of ac-DEVD-CHO; Western blot and densitometric analysis of a typical experiment out of 3 are reported. Densitometric analyses were normalized to Ponceau S staining.

**Figure 2:**
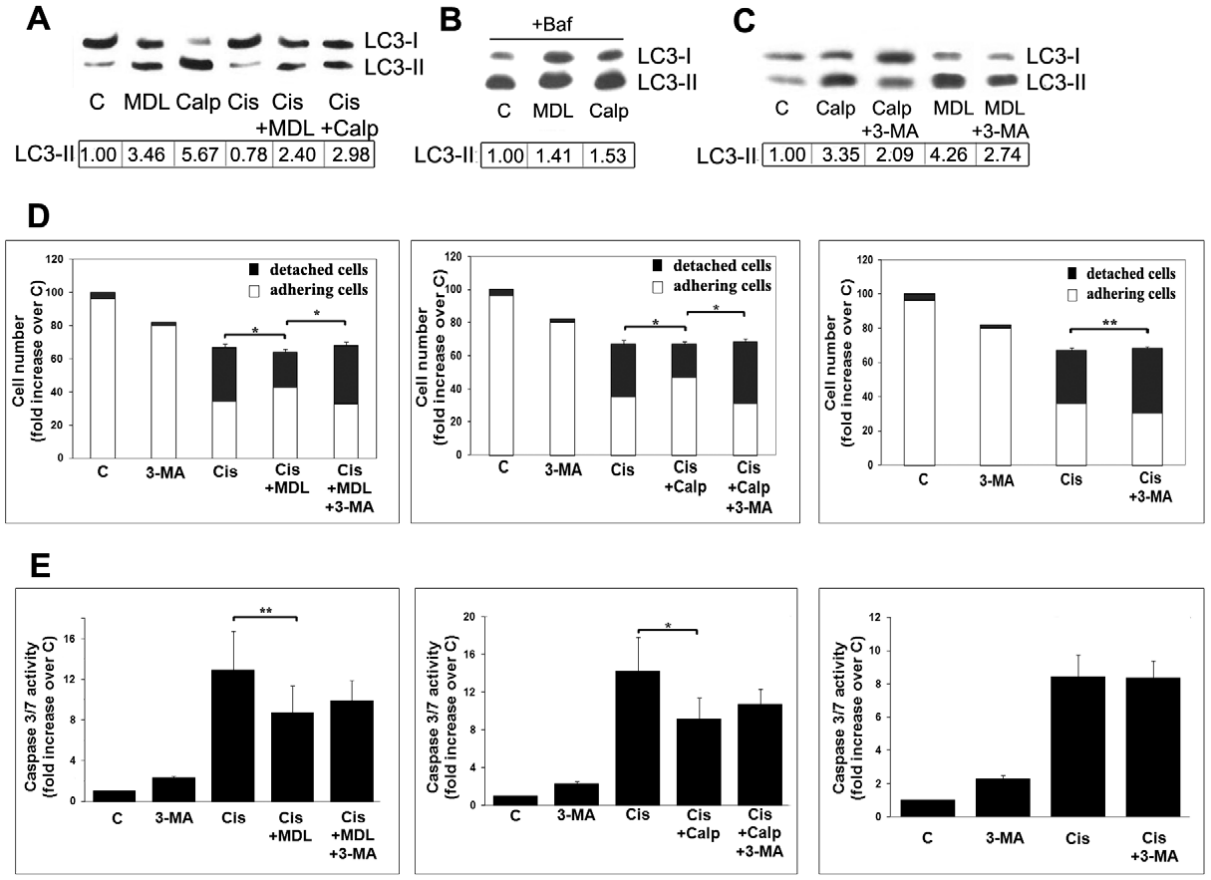
Calpains inhibitors induce autophagy and protect Me21 cells from cisplatin-induced apoptosis. Me21 melanoma cells were treated with 20 µM cisplatin for 24 h, in absence or presence of the calpains inhibitors MDL-28170 (MDL) and calpeptin (Calp) (40 µM both). (**A**) Western blot of LC3 and densitometric analysis of a typical experiment out of 4 are reported. Cisplatin-treated samples (single or combined treatment) refer to adhering cells. (**B**) Autophagic flux measured in the presence of bafilomycin A1, a vacuolar H+ ATPase inhibitor. Melanoma cells were treated for 22 hours with MDL-28170 or calpeptin, then exposed for further 2 hours to 200 nM bafilomycin A1. Western blot of LC3 and densitometric analysis of a typical experiment out of 3 are reported; in bafilomycin-treated cells half protein has been loaded. (**C**) Cells were treated with MDL-28170 and calpeptin for 24 hrs, in absence or presence of the autophagy inhibitor 3-methyladenine (3-MA); Western blot of LC3 and densitometric analysis of a typical experiment out of 4 are reported. Densitometric analyses were normalized to Ponceau S staining. (**D, E**) Cells were treated with cisplatin for 24 h, with or without the calpain inhibitors, MDL-28170 and calpeptin, and with or without 3-MA. (**D**) Total cell numbers are presented as percentage over control cells; the columns also show the relative number of adhering cells (white) and floating dead cells (black); S.E. values and statistical analysis refer to detached cells. (**E**) caspase-3/-7 activity; data are reported as means ± S.E. of 4 to 8 experiments. *P<0.001 and **P<0.05.

We examined whether cisplatin treatment, while inducing apoptotic cell death, was capable of modulating the basal autophagic process in Me21 melanoma cells. A crucial step for elongation of autophagosomal membrane is the conjugation of cytosolic LC3-I form with phosphatidylethanolamine (PE), producing the lipidated LC3-II form; the amount of LC3-II is the most widely used biomarker of autophagosomes formation. As shown by Western blot analysis of LC3-II (Fig. 1A), in melanoma cells treated with cisplatin, autophagy decreases below the basal level of control cells, both in still viable adhering cells and even more in floating cells at 24 hours of treatment, when a notable apoptosis occurs (Fig. 2D and E). At 18–20 hours of treatment, when cell detachment begins, and at shorter times, no significant change of LC3-II levels is evident (not shown).

Interpretation of LC3 immunoblotting can be problematic [20], [23], particularly when the basal autophagy, usually at low levels, is further lowered by different experimental conditions. For this reason, we also measured LC3-II under conditions where the autophagic flux is exogenously blocked, i.e. by co-treating melanoma cells with chloroquine (CQ), a lysosomotropic agent which inhibits the fusion of autophagosomes with lysosomes. In cells treated with cisplatin *plus* CQ, LC3-II remains significantly lower than that of control cells treated with CQ (Fig. 1B), thus confirming that cisplatin is actually inhibiting the basal autophagy.

In cisplatin-treated cells, inhibition of autophagosomes formation is also accompanied by down-regulation of two crucial components of the autophagic machinery, i.e. Beclin-1 and Atg14. Beclin-1 is a core component of the Class III Phosphatidylinositol-3-Kinase/Vps34 complex I, which is essential for the phagophore nucleation step of autophagy. In this complex, Beclin-1 serves as platform for assembly of different proteins which modulate Vps34 activity. Among these proteins, Atg14 contributes to complex I construction and to its localization at the pre-autophagosomal membranes. A time-dependent decrease of Beclin-1 is observed during cisplatin treatment (Fig. 1C). Furthermore, a fragment of Beclin-1 (of approximately 45 kDa) appears in floating apoptotic cells at 24 hours of treatment. Since cisplatin cytotoxicity is tipically associated to caspase-3/-7 activation, as demonstrated previously [16] and in the present work (Fig. 2E), we co-treated Me21 cells with the inhibitor ac-DEVD-CHO of these caspases. In cells co-treated with ac-DEVD-CHO, the formation of such a fragment is prevented and the loss of full-length protein is spared (Fig. 1D), indicating that Beclin-1 is a proteolytic target of these caspases. Similarly, Atg14 is also cleaved in frankly apoptotic cells to form a fragment of approximately 40 kDa. In presence of the caspase-3/-7 inhibitor, Atg14 proteolysis is significantly prevented, and the full-length protein tends to recover (Fig. 1E). Moreover, an increase of LC3-II levels is also evident in ac-DEVD-CHO co-treatment (Fig. 1F). The cytotoxicity induced by cisplatin is not changed in the presence of ac-DEVD-CHO (43%±4 *vs* 40%±3 of dead cells, in cisplatin- *vs* cisplatin *plus* DEVD-CHO-treated cells). Similarly, we previously demonstrated that inhibition of caspase-3/-7 activity by ac-DEVD-CHO, though preventing the proteolytic cleavage of some molecules involved in the apoptotic machinery (i.e. Bcl-2 and Apaf-1), was not capable of abrogating other apoptosis-associated events, including poly(ADP-ribose) polymerase (PARP) and lamin B proteolysis, chromatin condensation/fragmentation and the eventual detachment of dying cells. Such lack of protection against cytotoxicity induced by cisplatin can be due to the redundancy of other effector caspases involved in the degradation phases of apoptosis, and/or to the major role played by calpains, proved to be activated earlier than and up-stream to caspase-3/-7 [16].

### Stimulation of autophagy by inhibitors of conventional calpains protects Me21 melanoma cells from cisplatin-induced apoptosis

**Figure 3:**
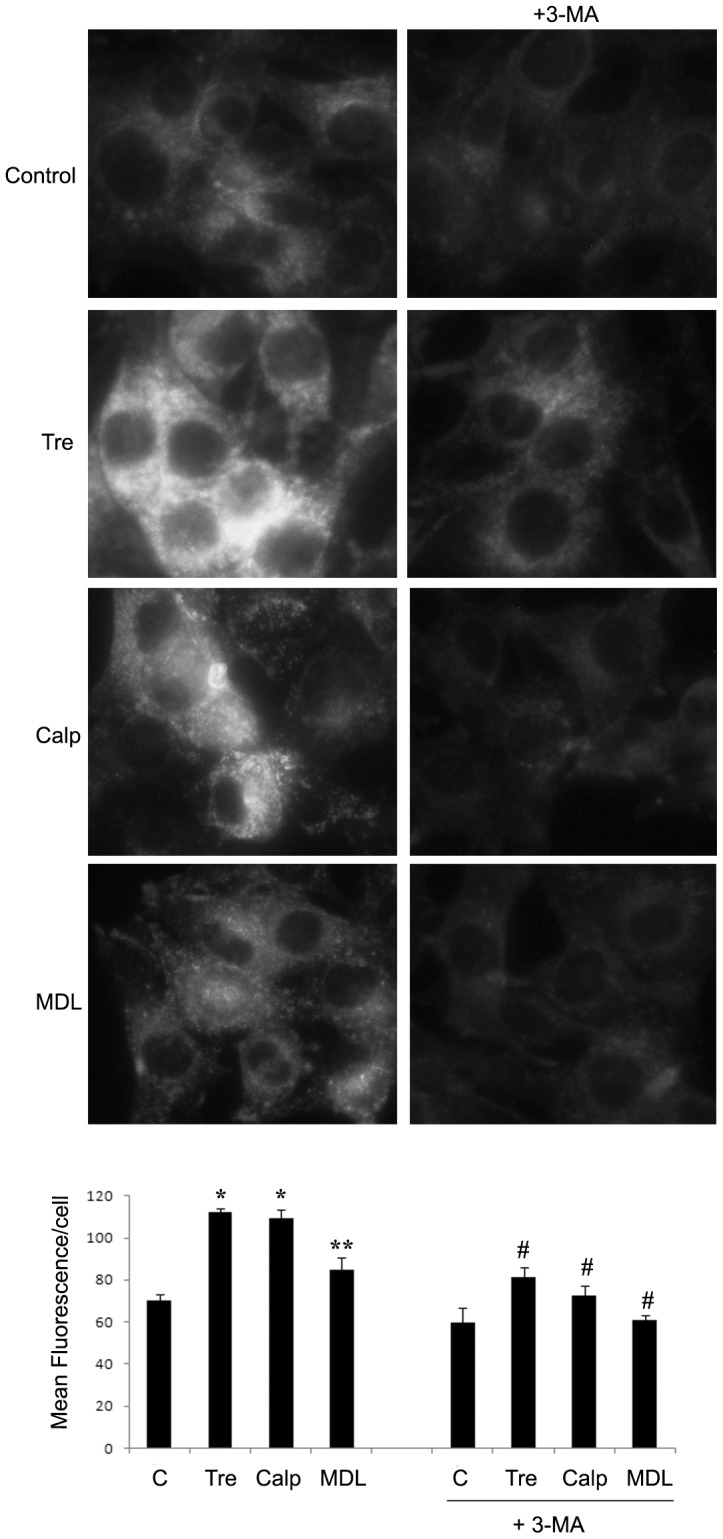
Autophagic vacuoles formation by different autophagy inducers. Me21 melanoma cells were treated for 24 hours with MDL-28170 (MDL), calpeptin (Calp) (40 µM both) and trehalose (Tre) (100 mM), in the presence or absence of 3-MA (4 mM). Cells were stained with monodansylcadaverine (MDC) and examined by fluorescence microscopy (Magnification, ×40). Quantitative data of MDC staining are reported as Mean Fluorescence/cell ± S.E. of 3 experiments. *P<0.01 and **P<0.05, compared to control cells; ^#^ *P*<0.001 compared to samples without 3-MA.

As we previously demonstrated in a similar experimental setting [16], in melanoma cells cisplatin treatment puts into motion the activation of conventional calpains, and two pharmacological inhibitors of these calpains (MDL-28170 and calpeptin), although not affecting the cytostatic effect of cisplatin, significantly protect from apoptotic cell death (Fig 2D and E), evaluated as cell detachment and caspase-3/-7 activity. We next asked whether such a protection could be in some way related to the autophagic machinery. Two lines of findings suggest that such a relationship does exist. First, both these calpain inhibitors induce a remarkable autophagic response in control cells, as revealed by the increased LC3-II levels (Fig. 2A). It is interesting to note that, similarly to the effect of cisplatin treatment on basal autophagy, also in the presence of these exogenous autophagy inducers, the apoptotic machinery triggered by cisplatin markedly counteracts the autophagic response, as revealed by the lowered LC3-II levels (Fig. 2A). The stimulation of autophagy by calpains inhibitors is confirmed by the autophagic flux blocker, bafilomycin A1 (an inhibitor of vacuolar ATPases, that alters the lysosomal pH), which maintains LC3-II levels in calpain inhibitors-treated cells higher that in control cells (Fig. 2B). Autophagy induction is also revealed by staining cells with monodansylcadaverine (MDC). Since MDC accumulates in mature autophagic vacuoles, such as autophagolysosomes, but not in early endosome compartment, MDC staining is commonly used to detect autophagic vacuoles. As shown in Fig. 3, in control cells a faint staining is evident; in MDL-28170 and calpeptin treated cells there is an increase of fluorescence intensity, both as diffuse staining and punctuate pattern (the latter more evident at higher magnification), distributed within the cytoplasm or often accumulated in perinuclear regions. A second line of findings suggests that a mechanistic relationship between protection from cell death and autophagy stimulation exists: in fact, the co-treatment with the upstream autophagy inhibitor 3-methyladenine (3-MA) (acting through class III PI3K inhibition [24]), while decreasing LC3-II levels (Fig. 2C) and MDC staining (Fig. 3), fully restores cell detachment (Fig. 2D). Also in the absence of any exogenous autophagy inducers, a mild increase of cell detachment is constantly observed in the co-treatment with cisplatin *plus* 3-MA. In all samples, such an increased cell detachment doesn't match with a comparable increase of caspase-3/-7 activity (Fig. 2E), suggesting a contribution of cell death achieved through a mode different from apoptosis or, at least, through a caspase-3/-7-independent mechanism.

### Stimulation of autophagy by trehalose protects Me21 melanoma cells from cisplatin-induced apoptosis and inhibits calpain activity

**Figure 4:**
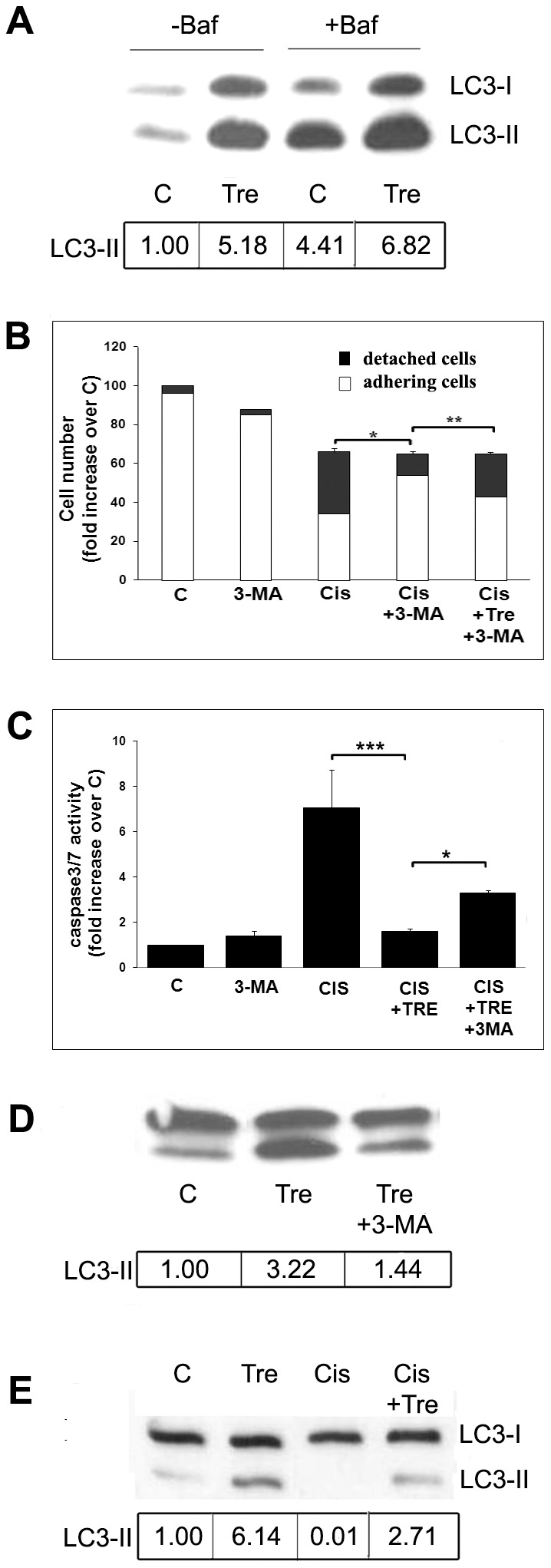
Trehalose induces autophagy and protects Me21 cells from cisplatin-induced apoptosis. (**A**) Me21 melanoma cells were treated with 100 mM trehalose (Tre) for 22 hours, then exposed to 200 nM bafilomycin A1 (Baf) for further 2 hours. Western blot of LC3 and LC3-II densitometric analysis of a typical experiment out of 3 are reported; in bafilomycin-treated cells half protein has been loaded. (**B, C**) Cells were treated with cisplatin for 24 h, with or without trehalose, and with or without 3-MA; (**B**) total cell numbers are presented as percentage over control cells; the columns also show the relative number of adhering cells (white) and floating dead cells (black); S.E. values and statistical analysis refer to detached cells; (**C**) caspase-3/-7 activity; data are reported as means ± S.E. of 4 to 5 experiments. *P<0.001,**P<0.01, and ***P<0.05. (**D**) Control cells were treated with 100 mM trehalose for 24 h, with or without the autophagy inhibitor, 3-methyladenine (3-MA); Western blot of LC3 and densitometric analysis of a typical experiment out of 5 are reported. (**E**) Cells were treated for 24 hours with cisplatin with or without 100 mM trehalose; cisplatin-treated samples (single or combined treatment) refer to adhering cells. Western blot of LC3 and LC3-II densitometric analysis of a typical experiment out of 4 are reported. Densitometric analyses were normalized to Ponceau S staining.

To confirm the protective role of autophagy as a more general mechanism in melanoma cells, we treated cells with a calpain-unrelated compound, that is the natural disaccharide trehalose [25]. This compound proves to be an efficient autophagy inducer in Me21 melanoma cells, as revealed by the increased LC3-II levels, which are confirmed by using the autophagic flux blocker bafilomycin A1 (Fig. 4A), and by the increased MDC staining (Fig. 3). Trehalose, although not affecting the cytostatic effect of cisplatin, affords a remarkable protection against cisplatin-induced cell death, evaluated as cell detachment and caspase-3/-7 activity (Fig. 4B and C). Similarly to what happens on calpains inhibitors, the inhibition of autophagy by 3-MA, while decreasing LC3-II levels (Fig. 4D) and MDC staining (Fig. 3), is capable of reversing the protective effect of trehalose, significantly on cell detachment and, to a lesser extent, on caspase-3/-7 activation (Fig. 4B and C).

**Figure 5:**
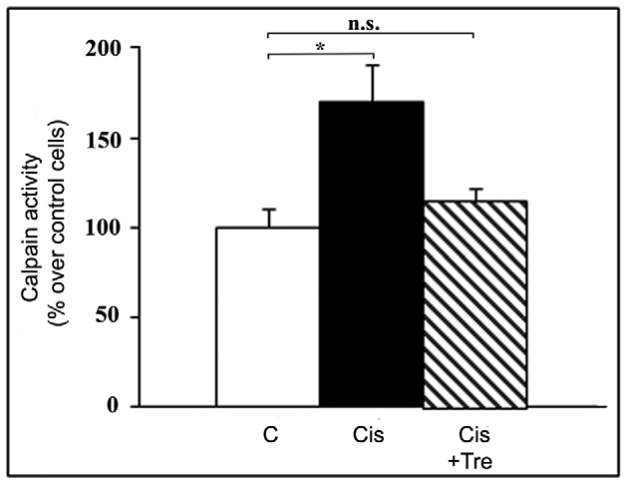
Autophagy inducer trehalose abrogates cisplatin-induced calpain activation in Me21 cells. Me21 melanoma cells were treated for 24 hours with cisplatin (Cis), with or without 100 mM trehalose (Tre); calpain activity was measured in cell lysates (adhering+floating cells) in the presence of the fluorogenic substrate *succ*-Leu-Tyr-AMC (200 µM). Results (expressed as percentage on control cells at 0 time) are given as means ± S.E. of 4 to 6 experiments. *P<0.05; n.s. = not significant.

The experiments carried out by using trehalose give two further informations: first, also with this autophagy inducer, cisplatin treatment tends to oppose the trehalose-stimulated autophagic response, as revealed by the decreased LC3-II levels (Fig. 4E); second, and more interestingly, the autophagic response induced by trehalose is capable of abrogating the cisplatin-induced calpain activation (Fig. 5).

### Cisplatin interferes with the autophagic process, which acts as a survival mechanism in HT-144 melanoma cells

**Figure 6:**
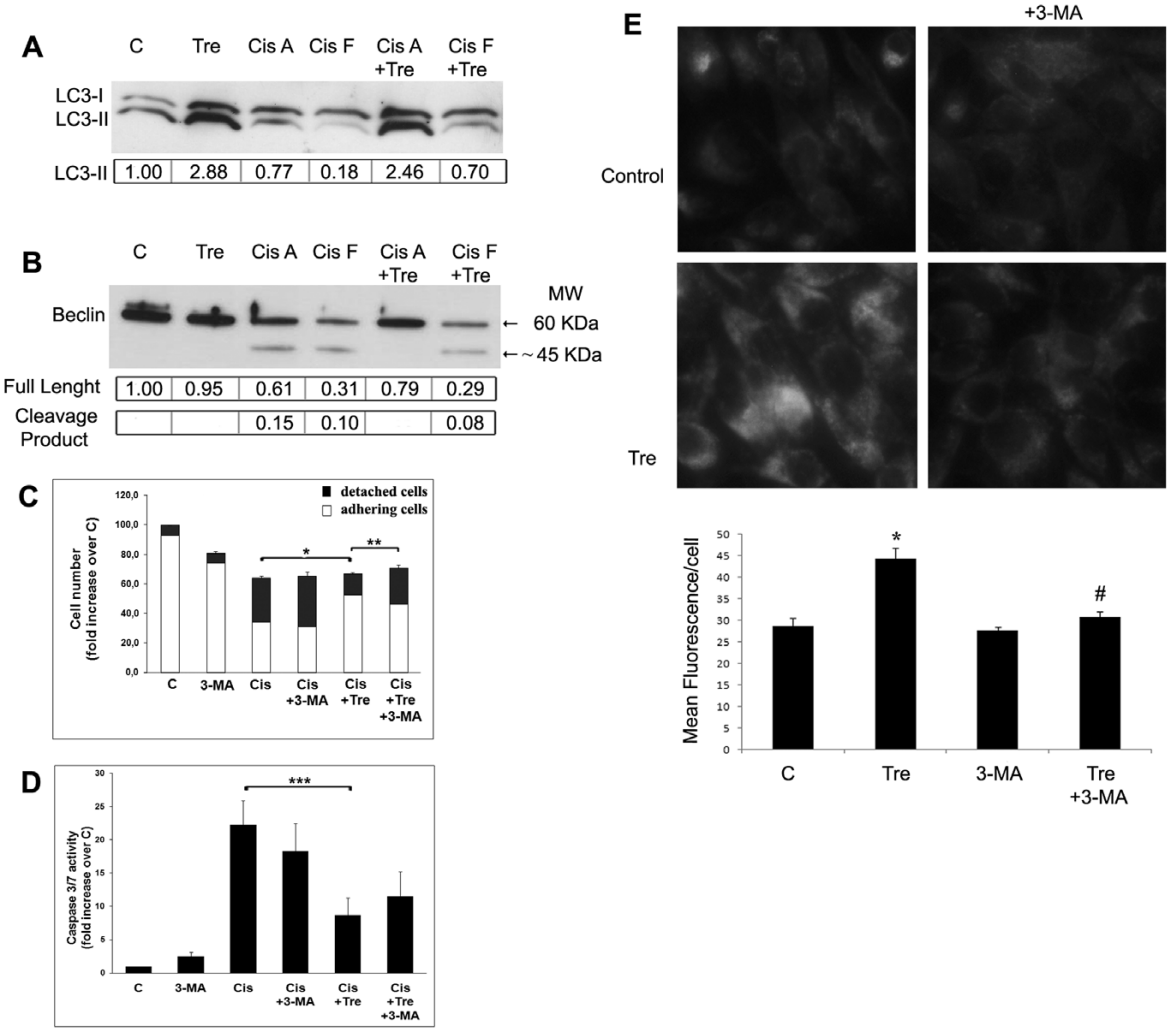
Autophagy protects HT-144 cells from cisplatin-induced cell death. HT-144 melanoma cells were treated for 24 hours with 15 µM cisplatin (Cis), with or without 100 mM trehalose (Tre). (**A**) Western blot of LC3 and LC3-II densitometric analysis of a typical experiment out of 3 are reported; (**B**) Western blot of Beclin-1 and densitometric analysis of a typical experiment out of 3 are reported. Densitometric analyses were normalized to Ponceau S staining. (**C, D**) Cells were treated with cisplatin for 24 h, with or without trehalose, and with or without 3-MA; (**C**) total cell numbers are presented as percentage over control cells; the columns also show the relative number of adhering cells (white) and floating dead cells (black); S.E. values and statistical analysis refer to detached cells; (**D**) caspase-3/-7 activity; data are reported as means ± S.E. of 3 experiments. *P<0.001,**P<0.005, and ***P<0.05. (**E**) HT-144 cells were treated for 24 hours with trehalose (Tre) (100 mM), in the presence or absence of 3-MA (4 mM). To reveal the formation of autophagic vacuoles, cells were stained with monodansylcadaverine (MDC) and examined by fluorescence microscopy (Magnification, ×40). Quantitative data of MDC staining are reported as Mean Fluorescence/cell ± S.E. of 3 experiments. *P<0.001 compared to control cells; ^#^ *P*<0.001 compared to samples without 3-MA.

Similarly to Me21 cells, cisplatin treatment is capable of modulating the basal autophagic process in HT-144 melanoma cells, as shown by the decreased levels of LC3-II (Fig. 6A, lanes 1,3,4). Moreover, the upstream autophagy player, Beclin-1, is also down-regulated by cisplatin treatment: a decrease of full-length protein is observed, along with the appearance of the same 45 kDa proteolytic fragment observed in Me21 cells, most likely due to caspase-3/-7-mediated cleavage (Fig. 6B, lanes 1,3,4). To assess the protective effect of autophagy stimulation on cisplatin-induced cell death, we performed experiments by co-treating HT-144 cells with cisplatin *plus* trehalose. The latter proves to be an efficient autophagy inducer in this cell line as well, as shown by the increased LC3-II levels (Fig. 6A) and MDC staining (Fig. 6E). The co-treatment with cisplatin *plus* trehalose, although not preventing the cytostatic effect of cisplatin, markedly decreases cell detachment and caspase-3/-7 activity (Fig. 6C and D). Interestingly, the protection against apoptosis is also effective on Beclin-1 consumption, which is partially spared in still adhering cells (Fig. 6B, lane 5). Similarly to Me21, in HT-144 cells the inhibition of trehalose-stimulated autophagy by 3-MA, as documented by the lowered MDC staining (Fig. 6E), is capable of reversing the protective effect of trehalose, significantly on cell detachment (Fig. 6C) and, to a lesser extent, on caspase-3/-7 activation (Fig. 6D).

### Inhibition of autophagy restores cell death also through plasma membrane damage

**Figure 7:**
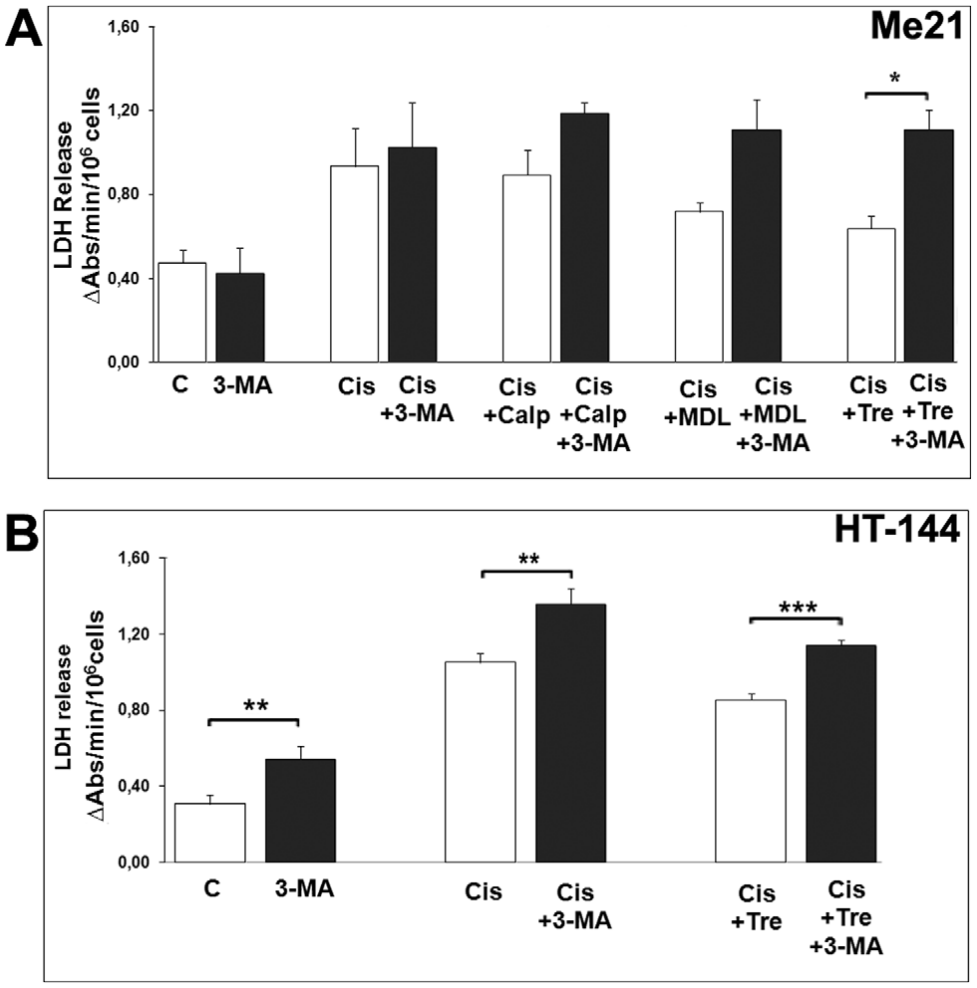
Autophagy inhibitor 3-MA enhances cisplatin-induced cell death through plasma membrane damage. Plasma membrane damage was evaluated by measuring LDH release in culture medium. (**A**) Me21 melanoma cells were treated for 24 hours with cisplatin, with or without the autophagy inducers MDL-28170, calpeptin (40 µM both) and trehalose (100 mM), in the presence or absence of the autophagy inhibitor 3-MA (4 mM). Data are expressed as means ± S.E. of 5 experiments. *P<0.005. (**B**) HT-144 melanoma cells were treated for 24 hours with cisplatin, with or without the autophagy inducers trehalose (100 mM), in the presence or absence of 3-MA (4 mM); data are expressed as means ± S.E. of 3 experiments. **P<0.05 and *P<0.001.

As above shown, in Me21 cells co-treated with autophagy inducers (MDL-28170, calpeptin and trehalose) *plus* cisplatin, as well as in HT-144 cells co-treated with trehalose *plus* cisplatin, the inhibition of autophagy by 3-MA restores significantly cell detachment but not significantly caspase-3/7 activity. Thus, we wondered if such 3-MA-mediated increase of cell death includes a contribution of non-apoptotic or caspase-independent cell damage. To verify this possibility, we measured the release of the cytosolic enzyme LDH in culture medium, as a marker of plasma membrane damage which occurs in different conditions of necrotic (or necrotic-like) cell death. As shown in Fig. 7, LDH release is constantly higher in the presence of 3-MA, suggesting that autophagy inhibition in cells undergoing both apoptosis and autophagy can put into motion alternative pathways of cytotoxicity, unexplored at the moment.

### Cisplatin down-regulates the autophagic process in A375 melanoma cells

**Figure 8:**
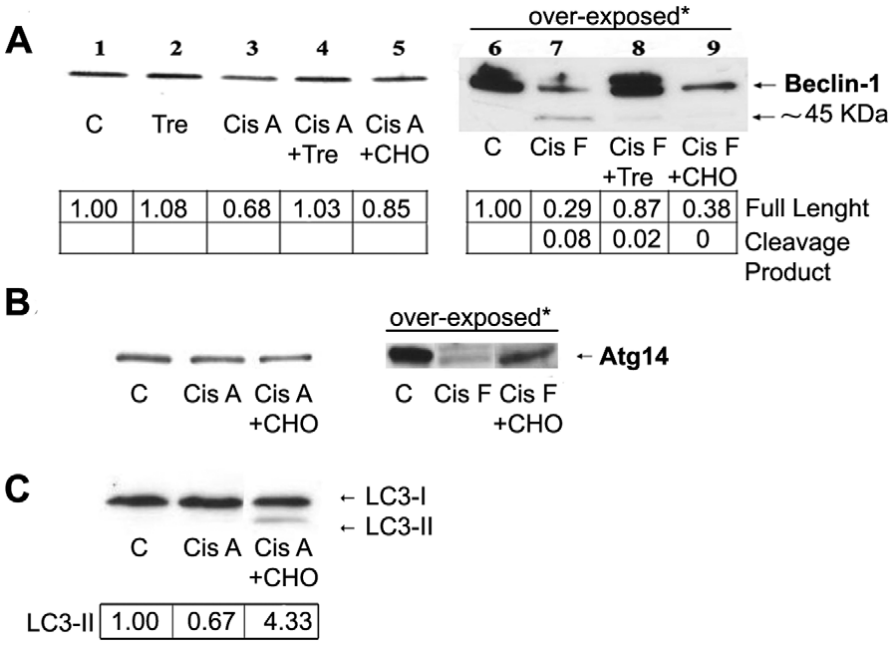
Cisplatin treatment inhibits the autophagic response in A375 cells. A375 melanoma cells were treated for 24 h with single or combined compounds: 60 mM trehalose (Tre), 20 µM cisplatin (Cis), 50 µM ac-DEVD-CHO (CHO); Cis A: cisplatin-treated adhering cells; Cis F: floating cells. Western blot of Beclin-1 and densitometric analysis of a typical experiment out of 3 are reported. *In order to better visualize the Beclin-1 proteolytic fragment, the photographic film was over-exposed. (**B**) Western blot of Atg14; a typical experiment out of 3 is reported. *In order to better visualize the full-length Atg14 recovery by ac-DEVD-CHO, the photographic film was over-exposed. (**C**) Western blot of LC3 and densitometric analysis of a typical experiment out of 3 are reported. Densitometric analyses were normalized to Ponceau S staining.

To confirm the results obtained in metastatic melanoma cells on non-metastatic melanoma cells, we also performed experiments in A375 cells; these cells respond to cisplatin to an extent of cell death comparable to Me21 cells, although calpain activation is minor (+10%±0.9, over control cells). Similarly to Me21 and HT-144 cells, in A375 cells the apoptotic machinery triggered by cisplatin inhibits autophagy, affecting the up-stream positive regulator, Beclin-1. Beclin-1 is down-regulated (Fig. 8A) in still adhering cells (lane 3), and much more in frankly apoptotic, floating cells (lane 7), where the 45 kDa fragment of Beclin-1 also appears. In cells co-treated with the inhibitor of caspase-3/-7, ac-DEVD-CHO, the full-length Beclin-1 is partially recovered and the formation of its fragment is prevented (lanes 5 and 9), confirming that Beclin-1 is a proteolytic target of these effector caspases. Another key component of the autophagic machinery, Atg14, is also down-regulated in cisplatin-treated apoptotic cells (Fig. 8B), and, similarly to Beclin-1, Atg14 loss is partially recovered by inhibiting caspase-3/-7. Prevention of Atg14 proteolysis by the caspase-3/-7 inhibitor cannot be evaluated also as decrease of fragment formation, because, differently from Me21 cells, Atg14 proteolytic fragment(s) are likely degraded quickly inside A375 cells. An increase of LC3-II levels is also evident in ac-DEVD-CHO co-treatment (Fig. 8C). Similarly to Me21 cells, the cytotoxicity induced by cisplatin is not changed in the presence of ac-DEVD-CHO (39%±3 *vs* 42%±5 of dead cells, in cisplatin- *vs* cisplatin *plus* ac-DEVD-CHO-treated cells).

### Trehalose-stimulated autophagy protects A375 melanoma cells from cisplatin-induced apoptosis

**Figure 9:**
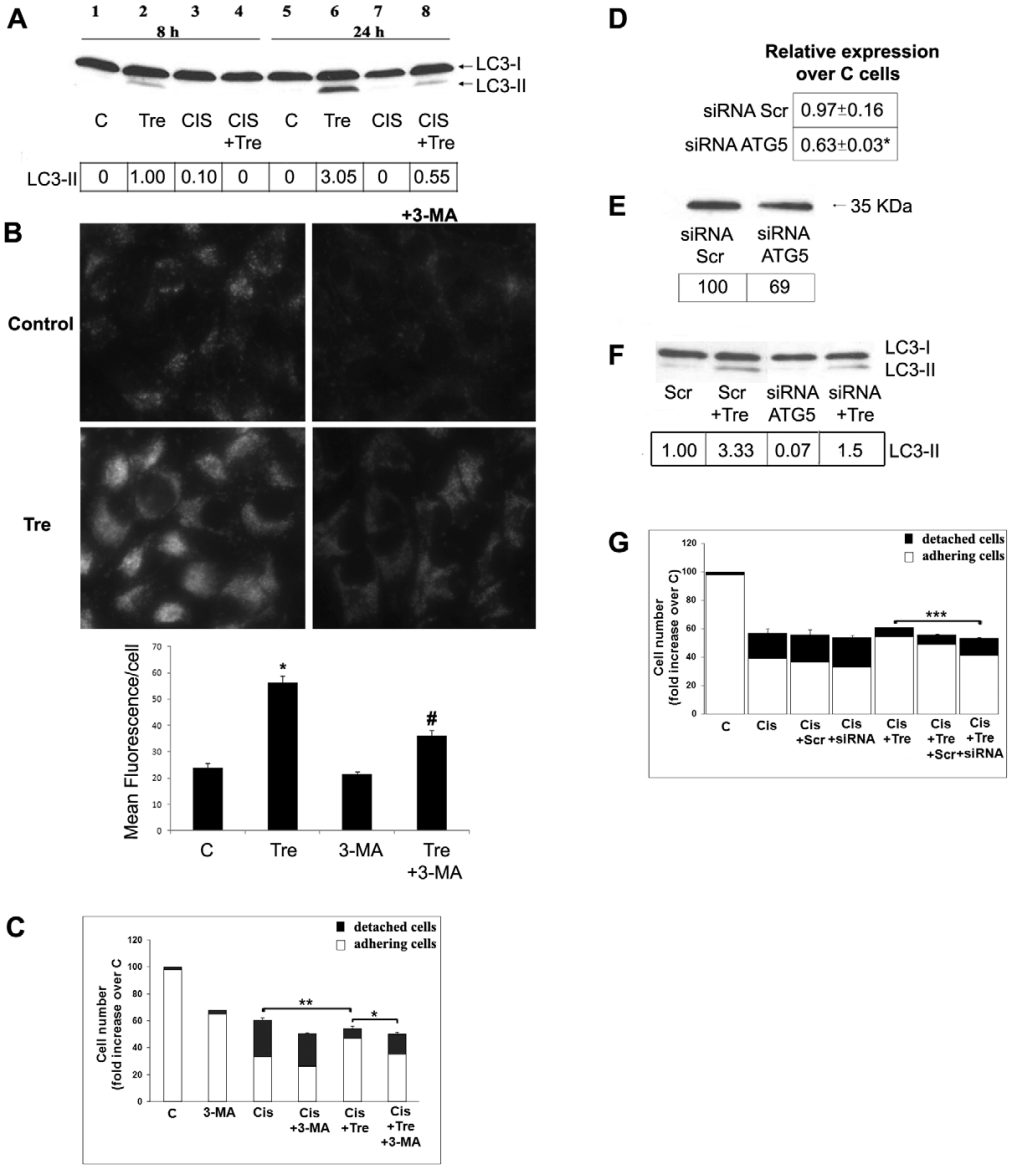
Inhibition of autophagy by *ATG5* silencing counteracts the protective effect of trehalose on cisplatin-induced apoptosis. (**A**) A375 melanoma cells were treated for 8 or 24 h with cisplatin, in the presence or absence of trehalose; Western blot of LC3 and LC3-II densitometric analysis of a typical experiment out of 3 are reported. (**B**) A375 cells were treated for 24 hours with trehalose (Tre) (100 mM), in the presence or absence of 3-MA (4 mM). Cells were stained with monodansylcadaverine (MDC) to evaluate autophagic vacuoles formation, and examined by fluorescence microscopy (Magnification, ×40). Quantitative data of MDC staining are reported as Mean Fluorescence/cell ± S.E. of 3 experiments. *P<0.01, compared to control cells; ^#^ *P*<0.01, compared to samples without 3-MA. (**C**) A375 cells were treated with cisplatin for 24 h, with or without trehalose, and with or without 3-MA. Total cell numbers are presented as percentage over control cells; the columns also show the relative number of adhering cells (white) and floating dead cells (black); S.E. values and statistical analysis refer to detached cells; data are reported as means ± S.E. of 3 experiments; **P<0.01 and *P<0.05; S.E. values and statistical analysis are related to detached cells. (**D**) A375 cells were transfected with scrambled siRNA or with ATG5 siRNA. The relative expression of ATG5 (mRNA) over non-transfected cells is reported; *P<0.05 compared to scrambled siRNA. (**E**) Western blot of Atg5 and densitometric analysis of a typical experiment out of 3 are reported. (**F**) Western blot of LC3 and densitometric analysis of a typical experiment out of 3 are reported. Densitometric analyses were normalized to Ponceau S staining. (**G**) Non-transfected cells, scrambled siRNA (Scr) or ATG5 siRNA (siRNA) transfected cells were treated with cisplatin for 24 h, with or without trehalose; Total cell numbers are presented as percentage over control cells; the columns also show the relative number of adhering cells (white) and floating dead cells (black); S.E. values and statistical analysis refer to detached cells. Data are reported as means ± S.E. of 3 experiments; ***P<0.001.

The inhibiting effect of cisplatin on the autophagic flux is difficult to evaluate in basal conditions, because of the very low or absent LC3-II level in control A375 cells. On the contrary, such inhibiting effect is well evident in conditions where autophagy is exogenously stimulated by trehalose. In fact, trehalose efficiently stimulates autophagy also in this cell line, as revealed by the time-dependent appearance of LC3-II (Fig. 9A, lane 2 and 6) and by MDC staining (Fig. 9B), and the treatment with cisplatin produces a remarkable decrease of LC3-II levels (Fig. 9A, lanes 4 and 8).

Trehalose-stimulated autophagy, although not affecting the cytostatic effect of cisplatin, affords a remarkable protection against cell death, here evaluated as cell detachment (Fig. 9C and G). Interestingly, the protection against apoptosis is also effective on Beclin-1 levels, which are fully restored (Fig. 8A, lanes 4 and 8). Similarly to Me21 and HT-144 cells, when the autophagic response induced by trehalose is pharmacologically inhibited by 3-MA (Fig. 9B), the protective effect is significantly reverted (Fig. 9C). In order to inhibit autophagy by means of a different tool, taking advantage from the high sensitivity of A375 cells to transfection procedures, we down-regulated *ATG5* gene expression by means of a specific siRNA. Although the extent of *ATG5* silencing was moderate, in terms of *ATG5* mRNA and protein (Fig. 9D and E), the trehalose-induced autophagic process is impaired, as revealed by the almost halved LC3-II level (Fig 9F). Such a decreased autophagic process is capable of significantly counteracting the protective effect of trehalose (Fig. 9G). A very minor cytostasis with no sign of toxicity was observed in scrambled-siRNA- and siRNA-transfected control cells.

### The mTOR-dependent autophagy inducer CCI-779 increases cisplatin-triggered apoptosis

**Figure 10:**
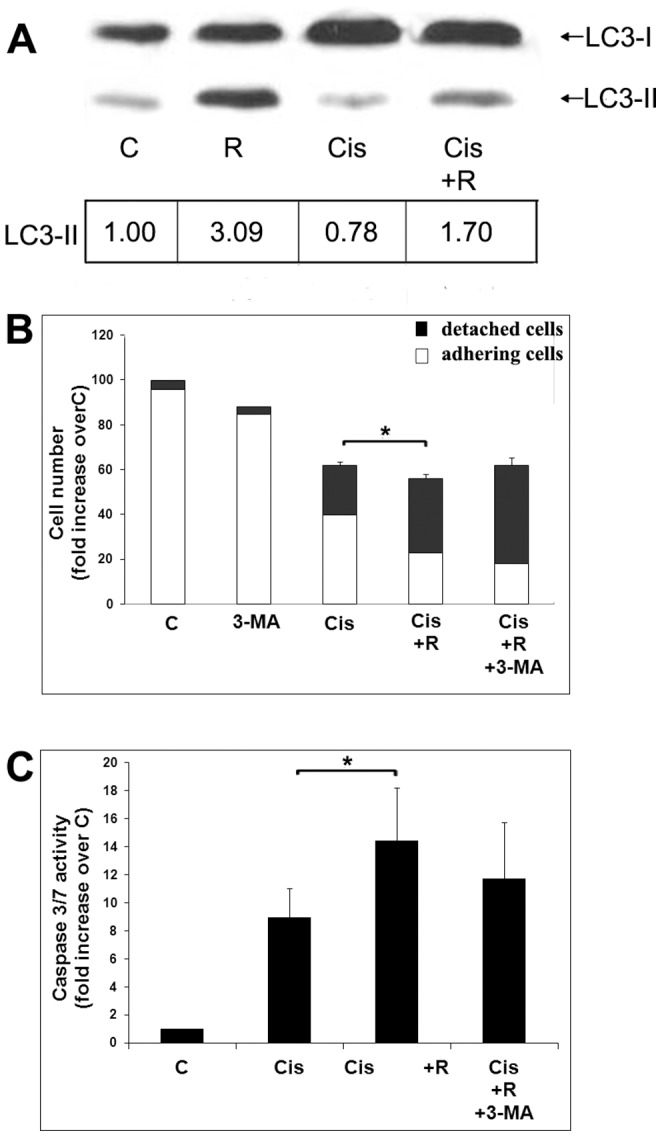
mTOR inhibitor CCI-779 (Rapamycin ester) induces autophagy but increases cisplatin-induced cell death. Me21 melanoma cells were treated for 24 hours with cisplatin, with or without 100 nM CCI-779 (R), in the presence or absence of 3-MA (4 mM). (**A**) Autophagy was evaluated by Western blot analysis of LC3. A typical experiment out of 4 is shown; densitometric analysis (normalized to Ponceau S staining) of LC3-II is reported below. (**B**) Total cell numbers are presented as percentage over control cells; the columns also show the relative number of adhering cells (white) and floating dead cells (black); S.E. values and statistical analysis refer to detached cells; (**C**) caspase-3/-7 activity. Data are reported as means ± S.E. of 3 experiments. *P<0.05.

To assess whether autophagy exerts a protective role on cisplatin-induced apoptosis irrespective of the nature of autophagy inducer, we co-treated Me21 melanoma cells with CCI-779, a more soluble ester of rapamycin, which is a well-known autophagic stimulus acting through mTOR inhibition [26]. In Me21 melanoma cells, CCI-779 is capable of inducing autophagy, as revealed by increased LC3-II levels, and also in this condition cisplatin treatment counteracts the autophagic response (Fig. 10A). While the treatment with CCI-779 alone, used in a wide range of concentrations (1–2,000 nM), exerts only a minor cytostatic effect on melanoma cells (data not shown), the co-treatment with cisplatin affords a remarkable synergistic effect on cell death produced by cisplatin alone, in terms of both cell detachment and caspase-3/-7 activity (Fig. 10B and C). In the treatment with cisplatin *plus* CCI-779 *plus* 3-MA, cell detachment is even more increased; in such triple poisoning, the extent of cell detachment does not mirror the extent of caspase-3/-7 activity, the latter being comparable to the co-treatment with cisplatin *plus* CCI-779 (Fig. 10B and C). According to our experience on this and other cellular models of apoptosis, such behaviour can be explained as a “physiological” degradation of highly activated caspases, which occurs when the rate of apoptotic cell death is remarkable (almost 80% in these experiments) and/or cell demise is long lasting.

## Discussion

A dose- and time-dependent induction of autophagy has been observed by others in different normal and tumor cells following cisplatin treatment [27]–[30]. Differently, in our experimental model of human melanoma cells which are sensitive to cisplatin, the drug inhibits the basal autophagic process. In Me21 melanoma cells, we have previously demonstrated [16], and here confirmed, that activation of conventional calpains 1 and 2 occurs along the apoptotic pathway. Since conventional calpains have been implicated in both pro- and anti-autophagic functions [12]–[15], in order to answer to the question whether and how activated calpains interplay with the autophagic response, we took advantage from two pharmacological inhibitors of calpains, MDL-28170 and calpeptin. In our study, autophagy is elicited when conventional calpains are inhibited, indicating that the basal calpain activity, and even more the increase of calpain activity triggered by cisplatin, are in charge of a negative control on the basal level of autophagy, as also suggested by others in different cellular contexts [13]–[15], [31]. Since cisplatin-mediated inhibition of autophagy still remains when calpain activity is abrogated by MDL-28170 and calpeptin, other mechanisms put into motion by cisplatin treatment must be responsible for the impairment of autophagy-related proteins. Among these mechanisms, the effector caspases 3 and 7 of the apoptotic machinery are the best candidates, as indicated by the proteolysis - reversed by DEVD-CHO, but not by calpain inhibitors (not shown) - of two key molecules of autophagy: Beclin-1, as also documented in other experimental models [32]–[34], and, as a novel finding to our knowledge, Atg14. Caspase-3/-7 inhibition also increases LC3-II levels, most likely due to the recovery of the above autophagy players or other Atg proteins acting up-stream of LC3 lipidation [34].

There is a growing body of evidence, obtained in different experimental settings, supporting the view that autophagy can be a constitutive metabolic state for invasive and metastatic phenotype of melanoma cells [35], [36], also related to vasculogenic mimicry and poor clinical prognosis of melanoma disease [37]. Other evidences also support the view that an enhanced autophagic activity, often induced by anticancer drugs themselves [38], can be an adaptive rescue mechanism that melanoma (and other tumor) cells use to escape from drug-induced apoptotic cell death [39]. Consistently, in the present study, either the basal autophagic activity (partially counteracted by cisplatin-triggered apoptotic machinery) or the stimulated autophagic response (afforded by calpains inhibitors and trehalose) prove to be protective towards cisplatin-mediated apoptosis, as also demonstrated by the fact that the upstream inhibition of autophagy constantly restores cell detachment and death. Indeed, as suggested by the increased LDH release, autophagy inhibition by 3-MA appears to restore the cisplatin-mediated cytotoxicity also through a necrotic-like mode of cell death, which is an interesting finding worth to be further explored.

The biological behaviour of melanoma cells to the rapamycin analogue, CCI-779, is peculiar if compared with the other autophagy inducers used in our study. The inefficacy of CCI-779 to protect melanoma cells from cisplatin-induced cell death, in spite of its capability to induce autophagy, can be related to the complexity of multiple pathways induced by mTOR inhibition, including up-regulation of pro-apoptotic proteins [40], and down-regulation of anti-apoptotic proteins [41]. It can be argued that, by inhibiting mTOR, the pro-survival contribution of the autophagic response can be overwhelmed by a pro-apoptotic contribution. In fact, in our experimental model, the frustrated attempt to escape from cell death by means of the autophagic response is disclosed by the effect of the autophagy inhibitor, 3-MA, which further increases the rate of cell death induced by cisplatin *plus* CCI-779. On the other hand, the similar response (i.e. protection from cisplatin-induced apoptosis) of melanoma cells to autophagy inducers, calpains inhibitors and trehalose, acting through mTOR-independent mechanisms [25], [42], indirectly suggests that autophagy can exert a pro-survival role through a pathway independent from (or down-stream of) mTOR.

Autophagy inhibition by activated calpains can derive from direct or indirect mechanisms. As a direct mechanism, it has been demonstrated that calpain-mediated cleavage of G_sα_ (α-subunit of heterotrimeric G-proteins), by enhancing the adenylyl cyclase activity of G_sα_, causes elevation of cAMP levels, which, in turn, inhibits autophagy [42]. Furthermore, numerous Atg proteins have been proved to be proteolytic targets of calpain 1 *in vitro* [34]. Among these, emphasis has been placed on calpain-mediated Atg5 proteolysis, also suggested as a mechanism switching cell fate from autophagy to apoptosis [43]. Possibly depending on different experimental settings, in melanoma cells of our study (data not shown), as well as in neuronal cells [42], such a cleavage does not occur. An indirect mechanism through which activated calpains could inhibit autophagy can be related to p53 status. A signaling pathway linking autophagy to cancer-associated p53 dysregulation has been suggested in the literature [44]: inhibition of p53 degradation, leading to its accumulation, is able to prevent the activation of autophagy; on the contrary, in diverse conditions of p53 pharmacological inhibition or genetic depletion/deletion, autophagy can be induced. Consistently with this scenario, in the same experimental model of the present study we have previously demonstrated [12] that a calpain-related p53 protein accumulation leads to apoptosis; the effector caspases of the apoptotic machinery could be in charge of inhibiting the autophagic response through a proteolytic impairment of key molecules, including Beclin-1 and Atg14, as we report here. Inversely, autophagy can be elicited when cisplatin-induced p53 up-regulation and apoptosis are both prevented or delayed by calpains inhibitors [16].

Along with the mechanisms through which activated calpains can repress autophagy, a related question is whether autophagy can affect calpain activity: in our study, trehalose-stimulated autophagy is capable of inhibiting calpain activity in Me21 cells. Since trehalose remarkably protects melanoma cells from apoptosis, it is conceivable that calpain activity inhibition is consequent to apoptosis inhibition; however, we can also envisage a sort of feedback loop, where autophagy can directly down-regulate calpain activity. Consistently, a similar relationship has been demonstrated in neuronal cells, where, in the opposite direction, inhibition of autophagy activates calpains [45].

**Figure 11:**
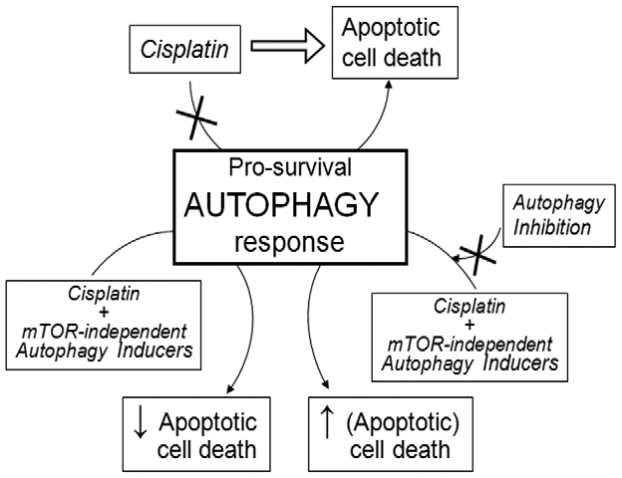
Outline of major findings and mechanisms proposed.

In conclusion, the major findings reported in the present study can be summarized according to the chart outlined in Fig. 11. In human melanoma cells, the death machinery triggered by cisplatin (including activation of calpains and caspases) is capable of inhibiting the basal autophagy, and such an inhibition, along with other mechanisms, contributes to apoptotic cell death. The pro-survival role of autophagy is confirmed by mTOR-independent pro-autophagic compounds, which protect from cisplatin-induced apoptosis. Such a protective mechanism is impaired by autophagy inhibitors (3-MA and *ATG5* silencing), which partially restore apoptotic (and non-apoptotic) cell death.

Further studies are needed to understand the signal transduction pathways involved in the reciprocal control between apoptotic cell death and autophagic response, and, based on this knowledge, to design more effective therapies for improving cell death [46]. Since the autophagic response proves to play a role as a self-protective mechanism in cisplatin-treated melanoma cells, it can be envisaged that the use of autophagy inhibitors may improve the efficacy of cisplatin-based chemotherapy for metastatic melanomas. However, the natural or acquired resistance to cisplatin, coupled to its severe side toxicity, is a major clinical limitation for this drug. Therefore, in a therapeutic perspective, the relationship between apoptotic cell death and autophagic response is worth to be investigated in metastatic melanoma cells treated with drugs (including dacarbazine and temozolomide) proved to be more relevant chemotherapeutic options.
