# Correction: Cisplatin-Induced Apoptosis Inhibits Autophagy, Which Acts as a Pro-Survival Mechanism in Human Melanoma Cells

**DOI:** 10.1371/annotation/8551e3d5-fdd5-413b-a253-170ba13b7525

**Published:** 2013-06-14

**Authors:** Barbara Del Bello, Marzia Toscano, Daniele Moretti, Emilia Maellaro

There was an error in affiliation 1 for all authors. The correct affiliation is: 1 Department of Pathophysiology, Experimental Medicine and Public Health, University of Siena, Siena, Italy, and Istituto Toscano Tumori (ITT), Italy.

